# The status of geo-environmental health in Mississippi: Application of spatiotemporal statistics to improve health and air quality

**DOI:** 10.3934/environsci.2018.4.273

**Published:** 2018-09-12

**Authors:** Swatantra R. Kethireddy, Grace A. Adegoye, Paul B. Tchounwou, Francis Tuluri, H. Anwar Ahmad, John H. Young, Lei Zhang

**Affiliations:** 1 Department of Natural Sciences and Environmental Health, Mississippi Valley State University, 14000 Highway 82 W, Itta Bena, MS 38941, USA; 2 College of Science, Engineering and Technology, Jackson State University, 1400 John R Lynch St., Jackson, MS 39217, USA; 3 Office of Health Data and Research, Mississippi State Department of Health, 570 East Woodrow Wilson Avenue, Jackson, MS 39215, USA

**Keywords:** environmental health, GIS, spatial analysis, asthma, air quality, regression analysis, spatiotemporal statistics, kriging, geocoding

## Abstract

Data enabled research with a spatial perspective may help to combat human diseases in an informed and cost-effective manner. Understanding the changing patterns of environmental degradation is essential to help in determining the health outcomes such as asthma of a community. In this research, Mississippi asthma-related prevalence data for 2003–2011 were analyzed using spatial statistical techniques in Geographic Information Systems. Geocoding by ZIP code, choropleth mapping, and hotspot analysis techniques were applied to map the spatial data. Disease rates were calculated for every ZIP code region from 2009 to 2011. The highest rates (4–5.5%) were found in Prairie in Monroe County for three consecutive years. Statistically significant hotspots were observed in urban regions of Jackson and Gulf port with steady increase near urban Jackson and the area between Jackson and meridian metropolis. For 2009–2011, spatial signatures of urban risk factors were found in dense population areas, which was confirmed from regression analysis of asthma patients with population data (linear increase of R^2^ = 0.648, as it reaches a population size of 3,5000 per ZIP code and the relationship decreased to 59% as the population size increased above 3,5000 to a maximum of 4,7000 per ZIP code). The observed correlation coefficient (*r*) between monthly mean O_3_ and asthma prevalence was moderately positive during 2009–2011 (*r* = 0.57). The regression model also indicated that 2011 annual PM_2.5_ has a statistically significant influence on the aggravation of the asthma cases (adjusted R-squared 0.93) and the 2011 PM_2.5_ depended on asthma per capita and poverty rate as well. The present study indicates that Jackson urban area and coastal Mississippi are to be observed for disease prevalence in future. The current results and GIS disease maps may be used by federal and state health authorities to identify at-risk populations and health advisory.

## 1. Introduction

Air pollution has become a burden on population health, as more than 2 million premature deaths every year can be attributed to the air pollution worldwide [1,2]. Environmental health is at an exciting stage. The 21^st^ century has witnessed the increased prevalence of respiratory-related health illnesses throughout the world [3], and the poor air quality is believed to be a significant cause for illnesses [4,5]. Air pollution is convincingly associated with many signs of asthma aggravation. These include pulmonary function decrements, increased bronchial hyperresponsiveness, visits to emergency departments, hospital admissions, increased medication use and symptom reporting, inflammatory changes, interactions between air pollution and allergen challenges, and immune system changes [6]. People living near roads with high traffic intensity are more likely to be affected by respiratory problems [7]. Many research studies have successfully established the relation between air pollution and its associated health effects [8–11]. It is less clear which pollutants are most responsible for causing respiratory illnesses, but traffic pollution might be playing a key role. Among the air pollutants, particles and O_3_ have the strongest associations [12]. Reports also convey that not only a single pollutant is responsible for causing respiratory-related health problems, but also a group of them (SO_2_, NO_2_, ozone (O_3_), and particulate matter (PM)) [13,14]. Around a quarter of the burden of disease is associated with environmental risk factors [15]. Risk factors for asthma may not be constant, and it is difficult to predict the exact cause. The risk may vary with time and by geography. The geography and subject’s spatial presence may play a major role in determining the environmental variables human being is exposed to [16,17]. They may also vary by sex, race, and age among individuals.

One in every 12 Americans has asthma, and its management is expensive, costing the U.S. $56 billion each year [18]. The access to health care is an important issue in the U.S. and other countries [19]. In the case of Mississippi (MS), data showed that the trend is similar to the rest of the U.S. [20]. Asthma is a growing epidemic in MS and in the U.S. as a whole [18,21]. MS has been affected by 36–44 extreme weather and climate events in the past 30 years and temperatures across the southeast region are expected to increase in future [22]. The consequence of increasing temperatures could be the formation of harmful air pollutants and allergens [23]. MS has been ranked at 17 among the 20 toxic industrially polluted states in the year 2010 [24]. A 2014 report suspected that outdoor air pollution, high amounts of pesticide exposures and energy sector might be serving as causative agents of asthma in Mississippians [25]. Dramatic improvements and developments in technology platforms may promise the integrated approach of understanding a spatial problem. The present study has laid a path to understanding the spatiotemporal dimensions of asthma in MS. Understanding its prevalence and outbreaks may help to make better decisions, save lives, and design healthy communities.

It is hypothesized that the spatiotemporal extent of asthma-related health problems is associated with the prevailing air pollution and asthma causes are distinctive for urban and rural areas. The objectives of this research are to: (1) assess and map the asthma rates in urban and rural MS, (2) highlight asthma health as a spatiotemporally significant disease and not simply from random events, (3) interpolate and model the air quality data concerning the particulate matter (PM_2.5_) and ground-level ozone (O_3_), and (4) analyze the statistical association of asthma to air pollution and poverty.

## 2. Materials and methods

### Study area

**Figure 1.**
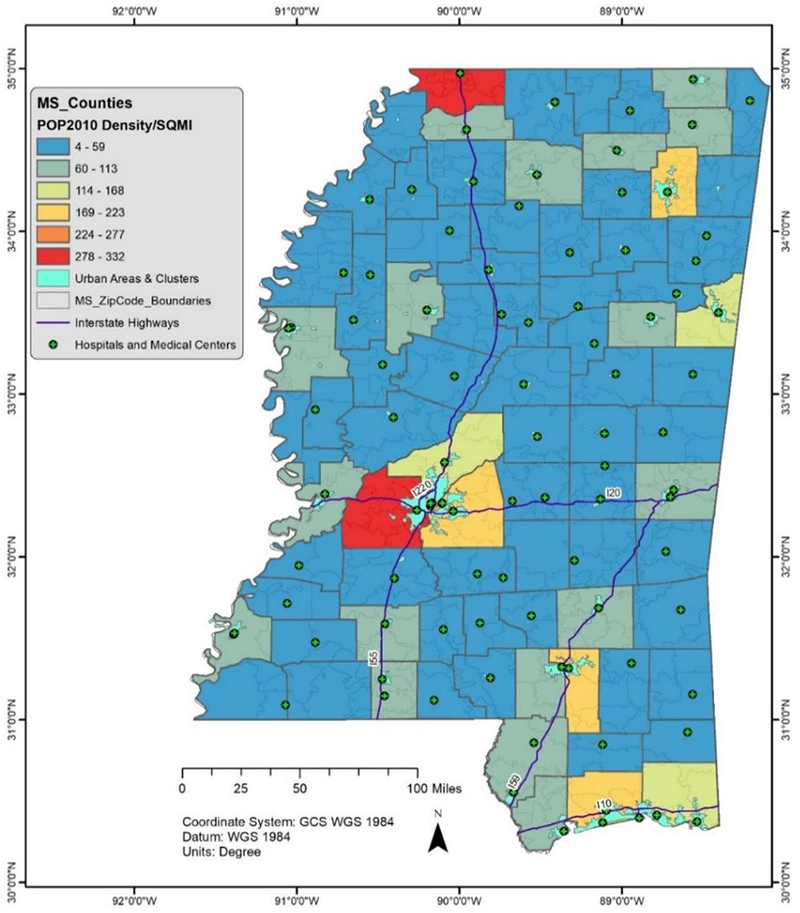
MS map showing the census 2010 population density, hospitals, and administrative units.

According to the 2010 census, the MS population is about 3 million people and has a density of 63 persons per square mile [26]. The primary economic activity of Mississippians is agriculture, fishing, mining, and timber. With a land area of 46,923.3 square miles, there are 82 counties, 5 urbanized areas, 64 urban clusters, 69 urban areas, and 424 ZIP Code tabulation areas in MS, see Figure 1 [26].

**Figure 2.**
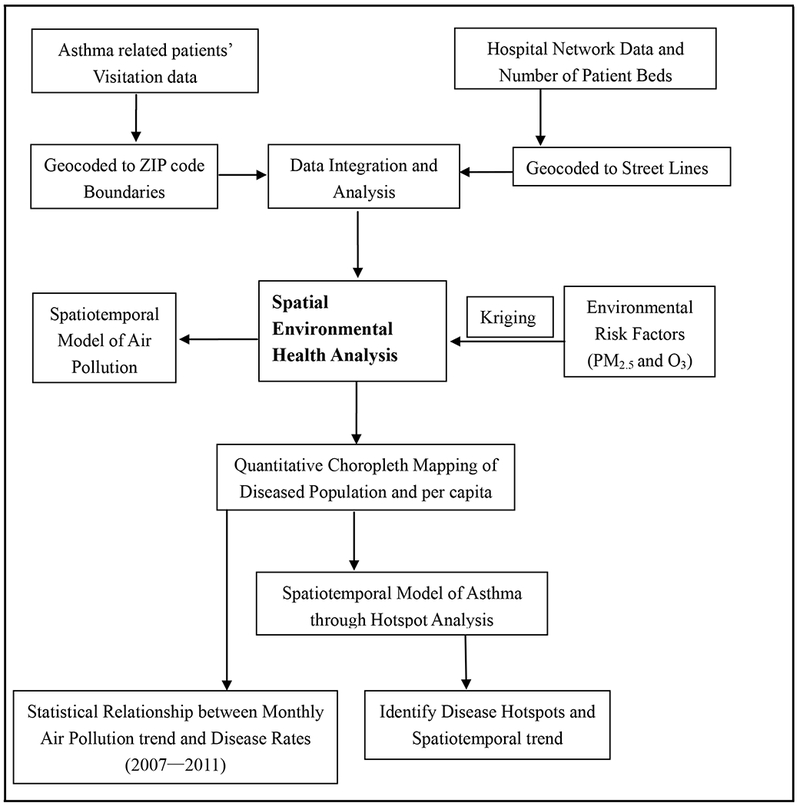
Overview of the research methods.

The following flowchart (Figure 2) explains the methods followed in processing and analyzing air quality and asthma health data. Geospatial statistical techniques were applied to the data of air quality and asthma patients visits. Asthma-related patient (inpatient, outpatient, and emergency visits) data were geocoded to ZIP Code boundaries, and hospital network data containing patient bed information were geocoded to street line data, (Figure 2). Later, the data were mapped using quantitative choropleth techniques in ArcGIS. Asthma per capita was calculated using census 2010 population data to understand the differences in urban and rural prevalence. Annual levels of PM_2.5_ and O_3_ were spatially interpolated by ordinary kriging method.

Asthma per capita data were further explored by spatial statistical techniques (hot spot analysis) to identify the hot and cold spots in urban and rural areas (Figure 2). Additionally, census population, and poverty data are taken as independent variables were statistically analyzed to understand their association with asthma. Time series models (or) seasonal cycles of asthma-related visits were also generated to reveal the temporal prevalence. The viable options for air quality metrics are to rely on the measurements from routine regulatory and deposition networks, intensive aircraft and ground-based field studies, radiosonde programs, satellite measurements, ground-based remote sensing networks, focused, fixed-site, and special purpose networks [27]. Table 1 below explains the data types used in the study, their sources and the spatial resolution at which the data were obtained.

These estimates reflect the economic characteristics of a geographic area over the entire five year period, and data are available for all geographic areas down to the census block group level [28]. The Census Bureau uses a set of dollar value thresholds that vary by family size and composition to determine who is in poverty. If a family’s total income is less than the dollar value of the appropriate threshold, then that family and every individual in it are considered to be in poverty. Similarly, if an unrelated individual’s total income is less than the appropriate threshold, then that individual is considered to be in poverty [29].

### 2.1. Geocoding of patient data and hospital addresses

Data were preprocessed in Microsoft access software and segregated by yearly format to be further analyzed using ArcGIS. The first step in the analysis process is geocoding, which assigns patient counts to the corresponding ZIP code. An address locator was created for ZIP code polygons. This tells the ArcMap which is reference data [30]. Once the address locator was created, patient data were geocoded by ZIP code. On average, 96% of geocoding accuracy was achieved for the patient data, the rest of 4% was an error because of the absence of ZIP code information. To visualize the patient counts by ZIP code, patients and ZIP code layers were spatially joined. Later, quantitative choropleth mapping technique was applied to show the number of patients by ZIP code. Data were classified into increasing interval classes based on equal interval method and a color scheme was assigned to each class. Each interval class had a range of patient numbers that increased with each class. Street line geocoding uses a foundation address database to match the addresses of health events or healthcare facilities. This method was chosen for hospitals because of the completeness of the hospital database for address and the method places points at an accurate location on the ground.

An address locator for street lines was created and the hospitals were geocoded to streets, and the fields containing latitude and longitude values were added to the database. Output contained few errors in the form of unmatched addresses because of errors in hospital address database or in street line database. The unmatched addresses were examined and fixed case by case by an interactive re-matching process.

#### 2.1.1. Finding asthma rates by spatial aggregation

Once patient data were geocoded by ZIP codes, the patient counts were joined to the ZIP code polygon. Disease rates were then calculated by dividing the counts with a total population of that ZIP code area.

#### 2.1.2. Investigation of asthma patients using hotspot analysis

In the process of hot spot analysis, a powerful set of spatial statistical tools were used to look at the distribution of values associated with geographic features. The tools used in this part of analysis were (1) Data Management tools (Project and Copy Features), (2) Spatial Statistics (Collect Events, Hot Spot analysis), and kriging tool in spatial analyst extension. By default, patient data were using geographic coordinate system instead of the projected coordinate system. Project tool was applied, and the data were projected by the NAD_1983_UTM_Zone_15N projected coordinate system to preserve the distance. The data that fall within a ZIP code were aggregated by applying “Collect events tool” and the resulting feature contained an “ICount” field reflecting the number of patients in that ZIP code area. The aggregated feature class was used as input and ICount field was used for hot spot analysis.

#### 2.1.3. Establishing the spatial relationships

Hotspot analysis tool looks for the spatial relationships in the data of interest. A feature with a high value surrounded by the other features with high values is called statistically significant hot spot (red areas) and the feature with a low value surrounded by other features with low values is called statistically significant cold spot (blue areas). The default spatial relationship is “Fixed Distance Band” means the features neighboring to each other within a critical distance receive a higher weight in spatial computation and the features away from the critical distance have no influence, and the distance method is “Euclidean Distance”. Distance band was chosen by finding the appropriate scale of analysis. It is difficult to predict the optimal distance (the distance at which the spatial processes are most active and exhibit clusters) band based on what geographical extent these asthma rates are promoting clusters. To estimate an optimal distance band, “Incremental Spatial Autocorrelation tool” was used to find a distance band that reflects the maximum spatial autocorrelation. This tool runs the spatial autocorrelation at increasing distances and assigns a Z-score for the observed spatial autocorrelation and the Z-scores were plotted against the increasing distance. The peaks were observed, and the distance bands were selected based on the of ZIP code areas. Once the optimal distance bands were estimated, the values were used in hot spot analysis. The output of hot spot analysis is a feature class where each feature had been assigned a Z-score and a P-value. Later, kriging method was applied to hotspot feature class and a continuous raster surface of heat map was generated.

### 2.2. Analysis of air quality

During the period 2007–2011, air quality data were analyzed in relation to asthma rates. The two environmental factors that were studied in this research were (1) ground level O_3_ and (2) PM_2.5_. MS had been observing the air quality since many years with a sparse network of stationary ground monitoring stations (Supplementary Figure S1). Mississippi Department of Environmental Quality (MDEQ) is the only agency that operates the ambient air quality network in the state [31]. O_3_ monitoring starts in the month of March and continued until the end of October for each year. PM_2.5_ sampling was done every third day and continued throughout the year. Lack of monitoring station network at specific spatial and temporal intervals was the bottleneck for optimal data size, thus to improve the richness of spatial data and the model accuracy, data were collected from the neighboring states of Louisiana, Arkansas, Tennessee, and Alabama to be integrated into the geospatial statistical analysis. Sampling stations available for each year are varied by a range for each pollutant studied. From 2007 to 2011, the yearly numbers of sampling stations for O_3_ were 141, 89, 89, 95, and 97, and the stations for PM_2.5_ were 124, 119, 135, 138, and 131, respectively.

#### 2.2.1. Spatial interpolation

In this study, a geostatistical method called ordinary kriging was applied to estimate the values at unmeasured locations. The principles of geostatistics operate on two key tasks: (1) to uncover the dependency rules and (2) to make predictions. Kriging is based on semivariogram and covariance functions, and the prediction of unknown values [32]. This method not only predicts the values at unmeasured locations but also provides the measure of the accuracy of prediction [33,34].

The general formula is:

$$\hat{Z}\left(s_{0}\right)=\sum_{i=1}^{N}\lambda_{i}Z(s_{i})$$

where:

$\hat{Z}(s_{0})$ is the value to be predicted for location so;

*N* is the number of measured values;

λ_i_ are the weights assigned to each measured point;

*Z* (S_i_) is the observed value at the location S_i_.

#### 2.2.2. Ordinary Least Squares (OLS) regression analysis

For the period 2009–2011, data on asthma count by ZIP code and population size for the corresponding zip code were applied in regression analysis to understand the statistical dependency on population size. Other independent variables taken into the analysis are the data on children, older adults, patient counts of adjacent years, and annual PM_2.5_ interpolated values. An additional regression model was constructed on PM_2.5_ as a dependent variable, and the poverty rate, percent of older adults, and asthma per capita as independent variables. ZIP code population data from Esri appeared more reliable to apply in regression analysis, data were available for 2010, 2012, and estimated for 2009 and 2011. The reason for excluding other years (2007–2008) in regression analysis is that absence of population data at a zip code scale. Economic variable (poverty) data for a period of 2007–2011 were downloaded from the U.S. Census Bureau, which was integrated as an independent variable in multivariate regression analysis. Standard line plots were generated for each independent variable against asthma. Monthly values of environmental variables (O_3_ and PM_2.5_) were investigated by correlation analysis to identify the possible association with asthma.

## 3. Results

### 3.1. The geography of asthma prevalence

Spatial analysis indicated that the asthma-related patients have increased geographically over a decade. Largest numbers of patients were observed in urban regions, and the highest asthma rate was found in rural regions (Supplementary Figure S2). Evidence from statistically significant hot spots (northwest, southwest, northeast, and southcentral regions) of asthma rates indicated that the disease had taken a major turn between 2009 and 2011. Highest rates of asthma (4–5.5%) were observed in Prairie, Monroe County for the three consecutive years (Figure S2). Prairie, Monroe County (red spot on the map) appeared to be the victim of asthma with highest levels of observed prevalence for three consecutive years, 2009–2011. The areas of northwest and southcentral MS are also significant for asthma. Although a higher asthma prevalence is not seen in the coastal region populations (Figure S2), the number of patients is increased every year near Gulfport-Biloxi-Pascagoula because of increasing population [38].

**Figure 3.**
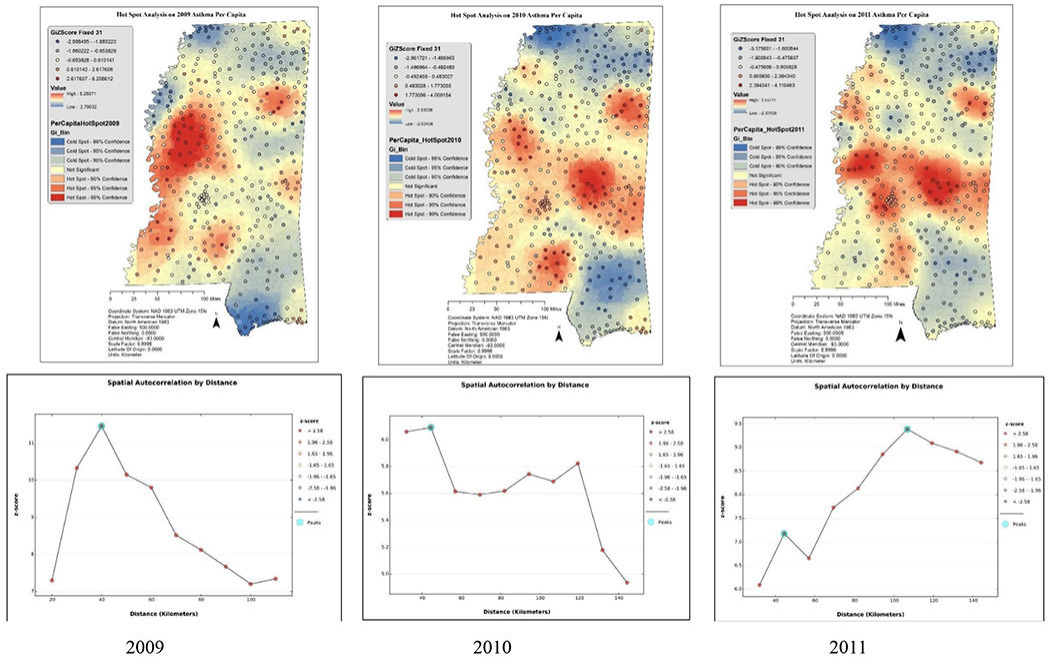
2009–2011 Hotspot analysis of asthma rates and spatial autocorrelation.

The maps of statistically significant hot spots and cold spots of asthma rates from 2009 to 2011 are presented in Figure 3. The summary of Z-scores and the distance observed at maximum autocorrelation were presented in Table 2. A high Z-score and small P value for a feature indicate a significant hotspot, a low negative Z score, and small P value indicates a significant cold spot [39]. Asthma health is not random in populations. It shows a significant spatial phenomenon indicated by the spatial clustering (Figure 3). The high Z-scores showed the Table 2 below indicate that the residuals are statistically significant and reject the null hypothesis that the asthma health is spatiotemporally random and spatially not autocorrelated.

### 3.2. Temporal pattern mining on asthma patient data

**Figure 4.**
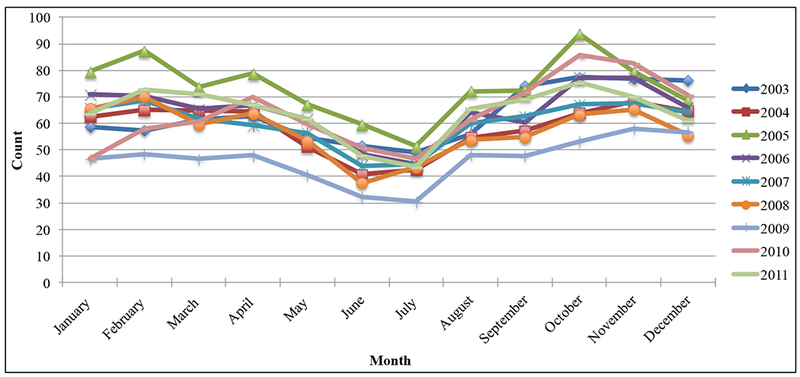
Average daily asthma-related inpatient, outpatient, and emergency visits.

**Figure 5.**
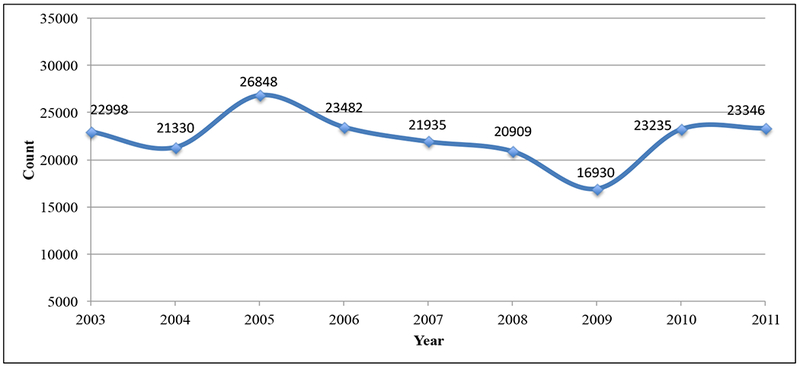
Total asthma-related visits for MS.

A steady and decreased trend in asthma-related visits was observed from 2005 to 2009 and the trend increased again beginning in 2010 (Figure 5). The highest daily average rates were observed in 2005 and the lowest in 2009 (Figure 4). For the remaining years, rates had fallen mostly between those two years. Average numbers of visits were lowest (30–51) in the months of June and July for all the analyzed data, increased beginning in August and peaked between the months of October and November.

From 2003 to 2011, the observed temporal pattern of asthma inpatient, outpatient, and emergency visit data is presented in Figure 4. The similarity in patients visits cycle, the pattern of timing, and common peaks were constant for all analyzed years, which indicated that the factors responsible for asthma exacerbations might be constant. These frequent exacerbations may be an indication of the greater severity of disease [42]. Asthma exacerbations peaked between September and October in each yearly cycle in MS. A related study investigated the similar phenomenon in North America and concluded that the increased consultations for childhood asthma every September were uniquely related to school return [43]. Children heading back to school had closer personal contact with many more children, therefore, increasing their exposure to viral and bacterial infections that could trigger an asthma attack [44]. A graph of total asthma-related patient visits is presented in Figure 5.

### 3.3. The geography of air pollution

**Figure 6.**
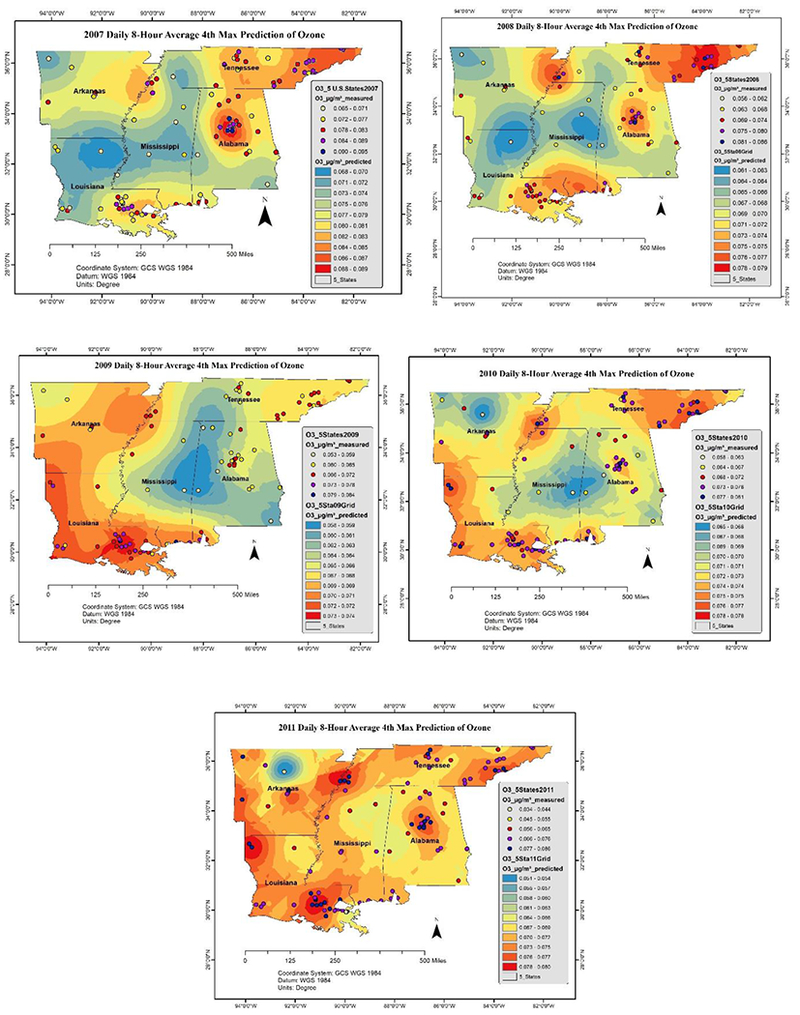
Daily 8-hour average 4^th^ max ground-level ozone (O_3_) pollution over MS.

**Figure 7.**
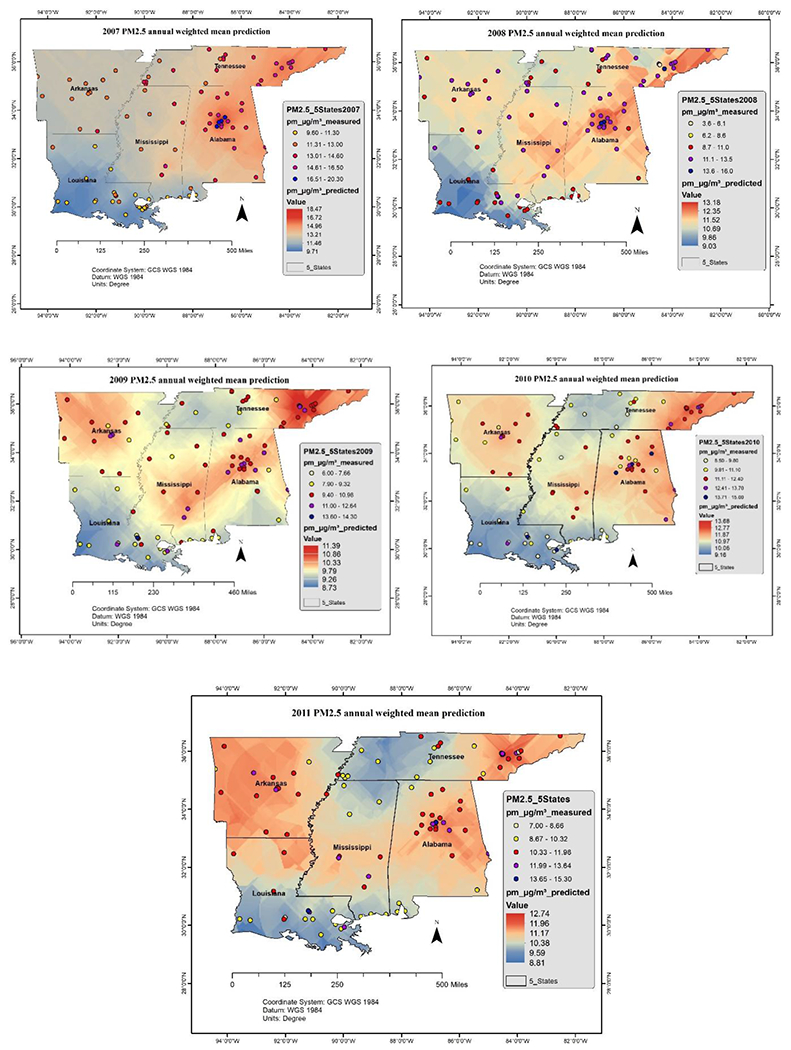
PM_2.5_ annual pollution levels over MS.

Ambient concentrations of air pollutants were visualized for five years (from 2007 to 2011). Spatiotemporal distribution was modeled by ordinary kriging method, refer the Figures 6 and 7.

The annual levels of O_3_ and PM_2.5_ predicted by ordinary Kriging model are presented in Figures 6 and 7 respectively. The highest levels of O_3_ (0.070–0.085 μg/m^3^) were observed in northwest, south, and southwest regions, and the highest levels of PM_2_._5_ (10–15 μg/m^3^) were observed in the southcentral and eastern regions. While U.S. EPA has revised the primary annual PM_2_.5 standard and tightened it to 12 micrograms per cubic meter (μg/m^3^) [45], the modeled values exceeded 12 μg/m^3^ during 2007 and 2008. The values remained close to the standard during 2010 and 2011 in the southcentral and central MS and posed a significant short-term and long-term threat to human health including premature mortality, increased hospital admissions and emergency department visits, and development of chronic respiratory diseases.

### 3.4. Statistical interpretation of asthma and population size

**Figure 8.**
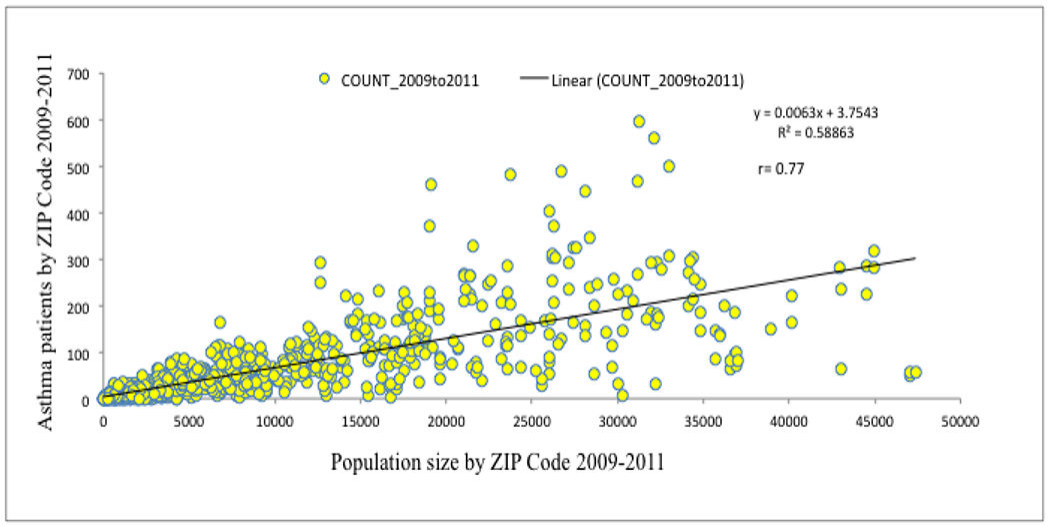
Association of asthma to population.

Between 2009 and 2011 (Figure 8), approximately 59% of asthma-related inpatient, outpatient, and emergency visits could be explained by the population size (coefficient of determination, R^2^ = 0.588 and correlation coefficient, *r* = 0.77). The relationship linearly increased to a maximum of 65%, R^2^ = 0.648 until it reached a population size of 3,5000 per zip code and the relationship decreased to 59% as the population size increased to a maximum of 47000 per ZIP code. This is a statistical signature about the contribution of other variables at densely populated (>3,5000/ZIP code) areas. Generally, higher populations were seen in urban regions, thus indicating the effects of urban risk factors in the prevalence of asthma.

Figure 8 provides an explanation about the dependency of asthma-related visits to corresponding population size at ZIP code spatial scale. Although a higher asthma prevalence was observed in rural MS, its association was proportionately distributed in the areas of ZIP codes with a population range of up to 35,000 indicating that the risk factors were constantly simple. As the population range increased above 35,000, the phenomenon became complex and was evidenced by the scattered data points in Figure 8, and also evident from population regression. It implies that complex risk factors were involved in the ZIP codes of urban areas with a larger population, which indicated that geography and urban settings are playing important roles. Research studies from many cities have documented that the urban heat island effects range from decreases in air quality, increased energy consumption, and alteration of the regional climate to direct effects on human health [41].

**Figure 9.**
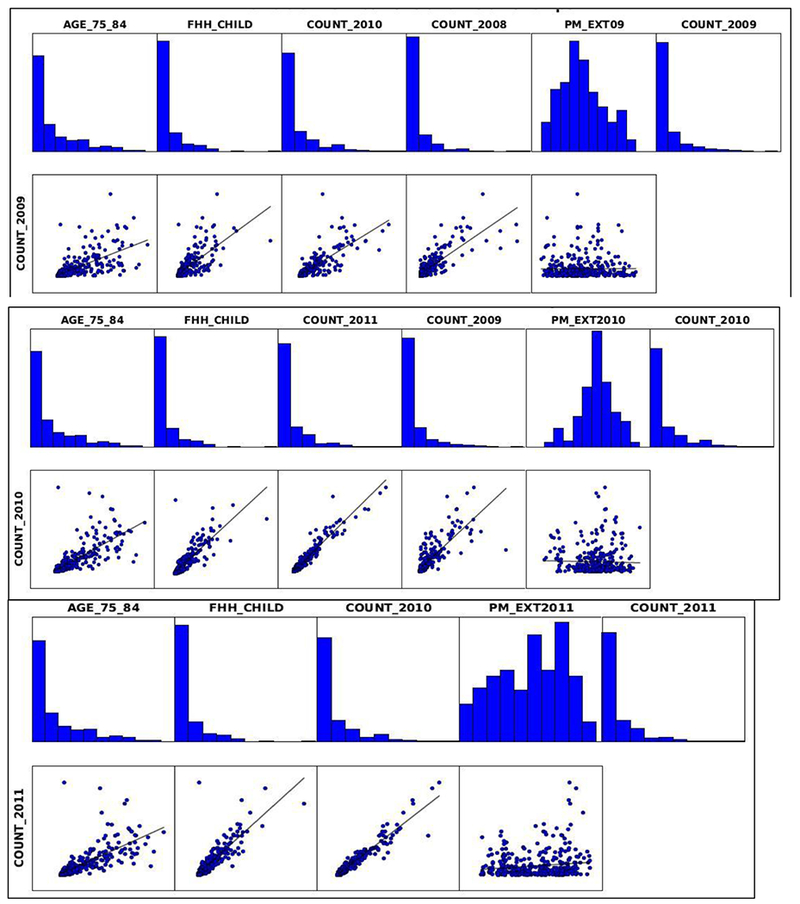
OLS Regression variable distributions and relationships for 2009, 2010, and 2011.

Based on the results in supplementary Table S1, the adjusted R-squared value was significantly improved (R-squared- 0.723, 0.934, and 0.930 for 2009, 2010, and 2011 respectively) from the model that used only the population data (R-squared- 0.588 in Figure 8). Within the population, the regression results explain the asthma dependency on children and older adults (Figure 9) and the probabilities with asterisk resemble the statistical significance. Coefficient [a] with a positive value indicates the positive relationship with asthma and a negative value indicates a negative relationship. Figure 9 provides the data for understanding the relationship of each variable with asthma count. The statistical significance in the Jarque-Bera statistics indicates a significant clustering in the fitted model, and the residuals from regression analysis are normally distributed, which means that the spatial pattern in the asthma health is not randomly generated. The asthma count of adjacent years is well correlated to the studied year, that means the health condition is mostly tied to the threshold levels from neighboring years indicating a consistent population that possessed the health condition.

**Figure 10.**
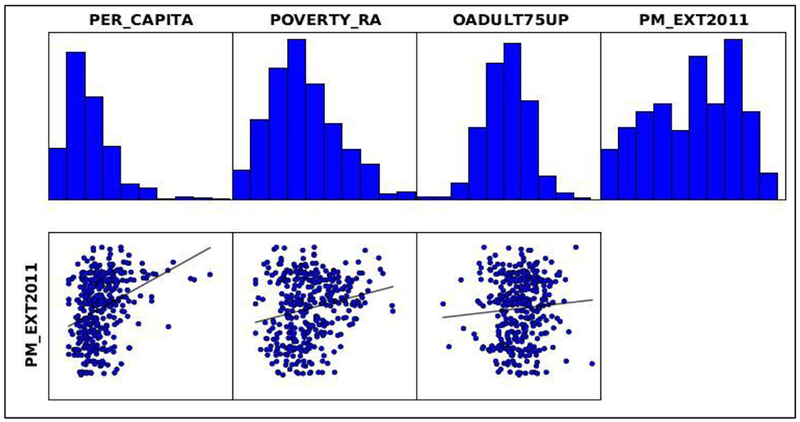
OLS Regression variable distributions and relationships 2011.

Poverty and asthma per capita can be explained by the PM_2.5_ for 2011, as shown in Figure 10 and coefficient [a] in supplementary Table S2. The relationship is positive from the positive sign on coefficient [a] and is further explained by the probability and robust probability columns, where an asterisk shows a statistical significance. Jarque-Bera statistic is also statistically significant, which means that the residuals from the fitted model did not show a random behavior, and the spatial pattern explains a significant clustering in the fitted variables. There is overwhelming evidence that exacerbations of asthma in terms of casualty attendances, hospital admissions, and deaths are related to poverty or to groups that are prevalent in poor sectors of the society [46]. Many published articles have discussed the poverty problem in the MS Delta [37,47–49].

**Figure 11.**
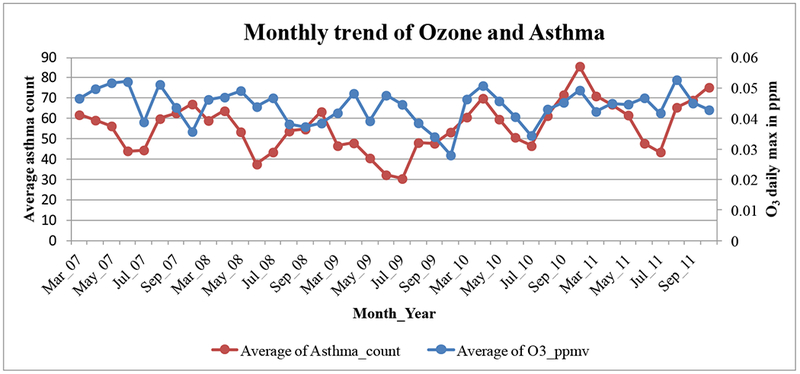
Correlation analysis of O_3_ and asthma.

Correlation analysis revealed an overall correlation coefficient (*r*) of 0.186 between monthly average levels of ozone and asthma count (Figure 11). The trend appeared to be synchronized in recent years from the second half of 2009. Since then, r increased to 0.572, indicating that there was a moderate positive correlation [35] between both the data.

**Figure 12.**
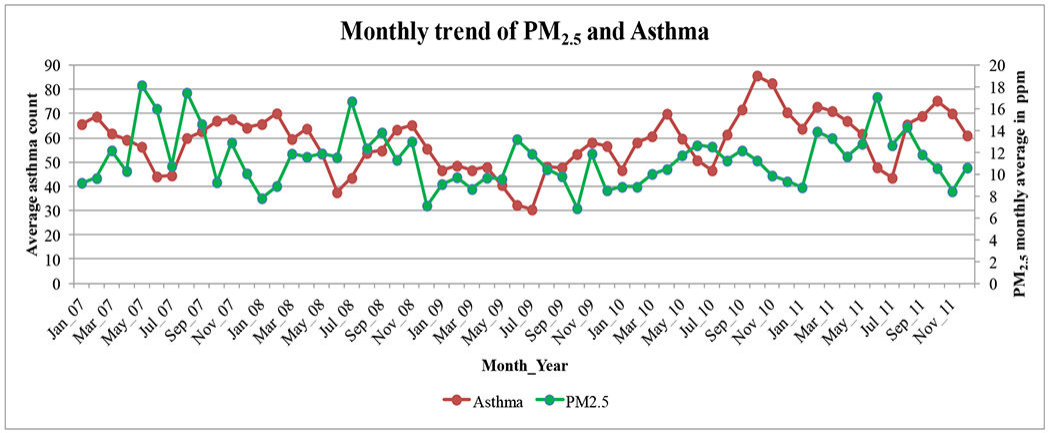
Correlation analysis of PM_2.5_ and asthma.

Correlation analysis did not reveal any significant association between PM_2.5_ and asthma (Figure 12), and there was a negligible negative correlation [35] of −0.125 observed between the data.

## 4. Discussion

The comprehensive geospatial approach undertaken in this research is first of its kind for addressing asthma problem in MS. Derivation of statistics of asthma at zip code level is unique in this research. The stakeholders, the public, and the administrators can understand about dissemination of disease and its affected regions by the help of produced maps and data. During 2009–2011, northwest MS has seen a continuous increase in the prevalence of asthma (Supplementary Figure S2 and Figure 3). This region is a floodplain of black alluvial fertile soils known for its agricultural heritage. Although poverty continues to prevail, farming remains the backbone for this region’s economy [36]. Many of the urban living patterns were seen in the rural delta region of MS such as the limited access to health care, pollution, and other environmental factors [37]. Large-scale interventions to reduce morbidity and mortality among rural patients with asthma in the U.S. have not been designed or implemented, despite rural Americans representing one of the most highly disadvantaged populations in the U.S [40]. Higher prevalence of asthma in rural MS could be an indication to support this fact. Asthma exacerbations were increasingly observed in the Jackson County region from 2006, urbanization might be an important risk factor in this region, which contributes to the accumulation of air pollutants by trapping heat.

Moderately significant associations were observed between monthly levels of O_3_ to asthma exacerbations, and the monthly associations were insignificant with PM_2.5_. This indicated that asthma populations are susceptible more towards seasonally varying environmental variables (O_3_), also for seasonal variability in asthma exacerbations almost similar trend is observed in every season for all the years studied. For 2011, there is a positive relationship observed and the annual PM_2.5_ levels depended on asthma per capita and poverty rate, as shown on Figure 10 and supplementary Table S2. The lag analysis might be useful to understand the health effects during short-term air quality-asthma events, but the current study had utilized the data from multiple years. The relationships with O_3_ were established only during the O_3_ sampling season from March to October in every year because the data were available only during this period.

## 5. Conclusion

Asthma is a complex health problem in MS, and distinctive spatial and temporal dimensions have been observed in asthma data. As hypothesized, its prevalence and risk factors were different in rural and urban areas. The risk factors were constantly simple in rural areas and more complex in urban areas. The highest rates (4–5.5%) were discovered in Prairie in Monroe County for three consecutive years. Statistically significant hotspots were observed in urban regions of Jackson and Gulf port, which mean the areas where a high number of patients are found in neighboring ZIP codes. It was visualized from hot spot analysis that the asthma rates have steadily increased near urban Jackson and the area between Jackson and Meridian metropolis. It is recommended that the Jackson urban area is monitored closely for disease prevalence in the future. The highest concentration levels of annual O_3_ (0.070–0.085 μg/m^3^) were observed in northwest, south, and southwest regions, and the highest levels of PM_2.5_ (10–15 μg/m^3^) were observed in the southcentral region. Statistical output indicated that 2009–2011 monthly O_3_ levels and 2011 annual PM_2_.s levels have a moderate influence on the aggravation of the asthma cases, as evidenced on Figures 10 and 11. As the adequate air quality data become available for MS, the statistical model performance would be further improved in the future studies. Between 2009 and 2011, spatial signatures of urban risk factors were found in zip code areas with dense populations. This was confirmed by the regression analysis of asthma patients with population size (R^2^ = 0.59). Resulting spatial data and information produced to provide a new insight into the management of environmental health data. The visualizations, info-graphics, and pictograms could be useful for decision making and asthma-related healthcare delivery in Mississippians. Statistically significant hotspots indicated that causative spatial processes are at work. Spatial analysis of asthma showed significant clusters in this delta region. Therefore, geographic clusters of poverty-asthma associations would be interesting to understand and may better visualize through regional scale analysis. Every year, vast amounts of population characteristics data are being generated by U.S. Census Bureau and are made available through its publicly available portals.

The environmental health GIS research results and disease maps generated from this research could potentially be useful to federal and state health authorities in treating the population at risk, as well as to develop and implement health advisories. Educational and awareness programs could be initiated, and proactive health needs may be delivered in targeted regions of MS (Delta, coastal, southcentral, and Prairie regions).

### Limitations

A network of sparse spatial observations for air quality is a limiting factor to obtain a trustworthy data at specific spatial intervals, and this is a backdrop for MS. Not all sources of data are available at a spatial resolution of ZIP codes. Intercensal population estimates for all zip codes were limited for this study. Because of this reason, disease rates were calculated for 2009, 2010, and 2011 by taking the 2010 population data as being constant for three years. Change of support problems (COSP) may result when trying to link the exposure data to the health outcome information because the two variables have inherently different scales. The disease is specific to an individual, but air quality varies over a continuum. Hence, these two different types of data not be always related in a way that permits a valid inference [51].

### Recommendations

The contribution of other confounding (agriculture, mobile source pollution, socio-economic, and racial) variables must be investigated to address the problem from its roots. Once these risk factors are identified, the public could be informed, and proactive measures can be taken to avoid the triggers and exposure. It may be a good decision to implement health education programs and health advisories in the MS Delta population. A street line data with information on updated routes and travel speed limits may be required to analyze the drive time for patients, which is a technique called network analysis that is used to better identify the underserved patients and deliver appropriate health care needs [52]. MDEQ may have to review plans for the observational network. A disease rate of around 4 to 6% in some zip codes is a significant issue to be addressed. Analysis of recent health data might reveal many insights on the potential conditions and geospatial distribution of asthmatics.

## Supplementary Material

Supplemental material

## Figures and Tables

**Table 1.** Data types, sources, and spatial resolution.

Data type	Source	Spatial resolution
Air quality (O_3_ and PM_2.5_)	U.S. EPAweb resources (http://www.epa.gov/airdata/)	Ground-based point locations
2010, 2012 population data	Esri ArcGIS online resources	ZIP code
Road network feature class	Esri street map premium	Street line
2003–2011 Asthma health data, hospitals, and beds	Mississippi State Department of Health (MSDH)	ZIP code
2007–2011 American Community Survey Poverty	Census Bureau’s American FactFinder (AFF) website (http://factfinder2.census.gov/faces/nav/jsf/pages/index.xhtml)	Zip code

**Table 2.** Global Moran’s I summary of asthma rates.

Year	First peak Distance in Kilometers	Z-score	Max peak Distance in Kilometers	Z-score
2009	40.00	11.45	40.00	11.45
2010	44.48	6.09	44.48	6.09
2011	44.47	7.17	106.79	9.38

## References

[1] World Health Organization. WHO Air quality guidelines for particulate matter, ozone, nitrogen dioxide and sulfur dioxide: global update 2005: summary of risk assessment. Geneva: World Health Organization, 2006 Available from: http://whqlibdoc.who.int/hq/2006/WHO%7B_%7DSDE%7B_%7DPHE%7B_%7DOEH%7B_%7D06.02%7B_%7Deng.pdf?ua=1

[2] Health Effects Institute. STATE OF GLOBAL AIR/2017, 2017 Available from: https://www.stateofglobalair.org/sites/default/files/SOGA2017_report.pdf

[3] Chan TC, Chen ML, Lin IF, (2009) Spatiotemporal analysis of air pollution and asthma patient visits in Taipei, Taiwan. Int JHealth Geogr 8: 26. 19419585 10.1186/1476-072X-8-26 PMC2694149

[4] Jerrett M, Burnett RT, Ma R, (2005) Spatial analysis of air pollution and mortality in Los Angeles. Epidemiology 16: 727–736. 16222161 10.1097/01.ede.0000181630.15826.7d

[5] Lemke LD, Lamerato LE, Xu X, (2014) Geospatial relationships of air pollution and acute asthma events across the Detroit-Windsor international border: Study design and preliminary results. J Expo Sci Environ Epidemiol 24: 346–357. 24220215 10.1038/jes.2013.78 PMC4063324

[6] Koenig JQ (1999) Air pollution and asthma. J Allergy Clin Immunol 104: 717–722. 10518814 10.1016/s0091-6749(99)70280-0

[7] D’Amato G (2002) Environmental urban factors (air pollution and allergens) and the rising trends in allergic respiratory diseases. Allergy 57: 30–33. 12144551 10.1034/j.1398-9995.57.s72.5.x

[8] Khatri SB, Holguin FC, Ryan PB, (2009) Association of ambient ozone exposure with airway inflammation and allergy in adults with asthma. J Asthma 46: 777–785. 19863280 PMC2837943

[9] Lin S, Liu X, Le LH, (2008) Chronic exposure to ambient ozone and asthma hospital admissions among children. Environ Health Perspect 116: 1725–1730. 19079727 10.1289/ehp.11184 PMC2599770

[10] Lu H, Qiu F, Cheng Y (2003) Temporal and Spatial Relationship of Ozone and Asthma. In: Esri International User Conference p.14 Available from: http://proceedings.esri.com/library/userconf/proc03/p0911.pdf

[11] Kumar N, Liang D, Cornelias A, (2013) Satellite-based PM concentrations and their application to COPD in Cleveland, OH. J Expo Sci Environ Epidemiol 23: 637–646. 24045428 10.1038/jes.2013.52 PMC3980441

[12] Schwartz J (2004) Air pollution and children’s health. Pediatrics 113: 1037–1043. 15060197

[13] D’Amato G, Liccardi G, D’Amato M, (2005) Environmental risk factors and allergic bronchial asthma. Clin Exp Allergy 35: 1113–1124. 16164436 10.1111/j.1365-2222.2005.02328.x

[14] D’Amato G, Cecchi L, D’Amato M, (2010) Urban air pollution and climate change as environmental risk factors of respiratory allergy: an update. J Investig Allergol Clin Immunol 20: 95–102. 20461963

[15] World Health Organization, Health is the key in motivating to solve environmental problems. World Health Organization, 2014.

[16] Davenhall B (2013) Geomedicine. Redlands, CA: Environmental Systems Research Institute, 32 Available from: http://www.esri.com/library/ebooks/geomedicine.pdf

[17] Davenhall B (2010) The Missing Component. ArcUser, 10–11. Available from: http://www.esri.com/news/arcuser/0110/files/geomedicine.pdf

[18] CDC (2010) Asthma’s Impact on the Nation, Data from the CDC National Asthma Control Program, 1–4.

[19] McLafferty S (2003) GIS and health care. Annu Rev Public Health 24: 25–42. 12668754 10.1146/annurev.publhealth.24.012902.141012

[20] MSDH (2011) 2011–2015 Mississippi State Asthma Plan.

[21] Roy SR, McGinty EE, Hayes SC, (2010) Regional and racial disparities in asthma hospitalizations in Mississippi. J Allergy Clin Immunol 125: 636–642. 20226297 10.1016/j.jaci.2009.11.046

[22] Carter LM, Jones JW, Berry L, (2014) Climate Change Impacts in the United States: The Third National Climate Assessment.

[23] Portier CJ, Thigpen TK, Carter SR, (2010) A Human Health Perspective on Climate Change: A Report Outlining the Research Needs on the Human Health Effects of Climate Change. Research Triangle Park, NC: Environmental Health Perspectives and the National Institute of Environmental Health Sciences.

[24] NRDC (2012) Toxic Power: How Power Plants Contaminate Our Air and States. Natural Resources Defense Council Environmental News.

[25] McMillin N, Listen to the lungs—The Daily Mississippian. The Daily Mississippian.

[26] Geography UCB, Guide to State and Local Geography—Mississippi, 2014 Available from: https://www.census.gov/geo/reference/guidestloc/st28_ms.html

[27] The National Science and Technology Council, Air Quality Observation Systems in the United States, 2013 Available from: https://obamawhitehouse.archives.gov/sites/default/files/.../air_quality_obs_2013.pdf

[28] U.S. Census Bureau (2008) A Compass for Understanding and Using american Community Survey Data: What General Data Users Need to Know. Washington DC: U.S. Government Printing Office.

[29] U.S. Census Bureau (2008) How Poverty is Calculated in the ACS.

[30] Kurland KS, Gorr WL (2012) GIS Tutorial for Health. Fourth edi. Redlands: Esri Press.

[31] MDEQ, Monitoring Network Plan—2014, 13.

[32] ESRI (2003) The principles of geostatistical analysis In: ArcGIS 9 Using ArcGIS Geostatistical Analyst. Redlands, CA: Esri Press, 49–78.

[33] Environmental Systems Research Institute I. How Kriging works—Help | ArcGIS for Desktop, 2016 Available from: http://desktop.arcgis.com/en/arcmap/10.3/tools/3d-analyst-toolbox/how-kriging-works.htm#

[34] Susanto F, de Souza P, He J (2016) Spatiotemporal Interpolation for Environmental Modelling. Sensors (Basel) 16 Available from: http://www.ncbi.nlm.nih.gov/pubmed/27509497 10.3390/s16081245 PMC5017410 27509497

[35] Mukaka MM (2012) Statistics corner: A guide to appropriate use of correlation coefficient in medical research. Malawi Med J 24: 69–71. 23638278 PMC3576830

[36] The Economist explains, Why are so many people leaving the Mississippi Delta? 2013 Available from: https://www.economist.com/blogs/economist-explains/2013/10/economist-explains-12

[37] Malik HU, Kumar K, Frieri M (2012) Minimal difference in the prevalence of asthma in the urban and rural environment. Clin Med Insights Pediatr 6: 33–39. 23641164 10.4137/CMPed.S9539 PMC3620776

[38] USA Today. Miss, coast shows modest population gains post-Katrina, 2011 Available from: http://usatoday30.usatoday.com/news/nation/census/2011-02-03-census-mississippi%7B_%7DN.htm

[39] CDC(2013) Introduction to Hotspot Analysis.

[40] Valet RS, Perry TT, Hartert TV (2009) Rural health disparities in asthma care and outcomes. J Allergy Clin Immunol 123: 1220–1225. 19233453 10.1016/j.jaci.2008.12.1131 PMC2738930

[41] Luvall J, Quattrochi D, Rickman D (2015) Boundary Layer (Atmospheric) and Air Pollution In: North GR, Pyle J, Zhang F, editors. Encyclopedia of Atmospheric Sciences. 2nd edition Elsevier Ltd, 310–318.

[42] Sears MR (2008) Epidemiology of asthma exacerbations. J Allergy Clin Immunol 122: 662–668. 19014756 10.1016/j.jaci.2008.08.003

[43] Sears MR, Johnston NW (2007) Understanding the September asthma epidemic. J Allergy Clin Immunol 120: 526–529. 17658590 10.1016/j.jaci.2007.05.047 PMC7172191

[44] Minnesota Department of Health, Asthma Hospitalizations Peak in September, 2008 Available from: http://www.health.state.mn.us/asthma/documents/08asthmahosppeaksept.pdf

[45] Federal Register (2013) National Ambient Air Quality Standards for Particulate Matter. 40 CFR Parts 50, 51, 52, 53 and 58 United States of America, 3086–3287.

[46] Rona RJ (2000) Asthma and poverty. Thorax 55: 239–244. 10679545 10.1136/thorax.55.3.239 PMC1745704

[47] Zheng J (2011) Shacks in the Mississippi Delta. Arkansas Rev A J Delta Stud 42: 30–33.

[48] Eudy RL (2009) Infant Mortality in the Lower Mississippi Delta: Geography, Poverty and Race. Matern Child Health J 13: 806–813. 18278546 10.1007/s10995-008-0311-y

[49] Abrokwa A, Chan A, Ha M (2010) Coverage Is Not Enough: Health Care Reform through the Lens of the Mississippi Delta. Kennedy Sch Rev Available from: https://www.highbeam.com/doc/1G1-247740034.html

[50] Seltenrich N (2014) Remote-Sensing Applications for Environmental Health Research. Environ Health Per sped 122: 268–275. 10.1289/ehp.122-A268 PMC4181909 25272250

[51] Gotway CA, Young LJ (2002) Combining incompatible spatial data. J Am Stat Assoc 97: 632–648.

[52] Pratt M, Moore H, Craig T (2014) Solving a Public Health Problem Using Location-Allocation. ArcUser, 56–59. Available from: http://www.esri.com/~/media/Files/Pdfs/news/arcuser/0614/solving-a-public-health-problem.pdf%5Cnhttp://www.esri.com/~/media/Files/Pdfs/news/arcuser/0614/summer-2014.pdf

