# Supplementary material for: The status of geo-environmental health in Mississippi: Application of spatiotemporal statistics to improve health and air quality
Source: AIMS Environ Sci. Author manuscript; Available in PMC 2018 Oct 25. (PMC6201236; doi:10.3934/environsci.2018.4.273)
Supplement: Supplemental material [file NIHMS989059-supplement-Supplemental_material.pdf]

Environmental Science

http://www.aimspress.com/journal/environmental

AIMS Environmental Science, 5(4): 273–293.
DOI: 10.3934/environsci.2018.4.273
Received: 15 March 2018
Accepted: 04 September 2018
Published: 12 September 2018

*Research article*

# The status of geo-environmental health in Mississippi: Application of spatiotemporal statistics to improve health and air quality

**Swatantra R. Kethireddy[1,]*, Grace A. Adegoye[1], Paul B. Tchounwou[2], Francis Tuluri[2], H. Anwar Ahmad[2], John H. Young[2] and Lei Zhang[3]**

[1] Department of Natural Sciences and Environmental Health, Mississippi Valley State University, 14000 Highway 82 W, Itta Bena, MS 38941, USA

[2] College of Science, Engineering and Technology, Jackson State University, 1400 John R Lynch St., Jackson, MS 39217, USA

[3] Office of Health Data and Research, Mississippi State Department of Health, 570 East Woodrow Wilson Avenue, Jackson, MS 39215, USA

* **Correspondence:** Email: swatantra.kethireddy@mvsu.edu; Tel: +16622543394; Fax: +16622543668.

## Supplementary

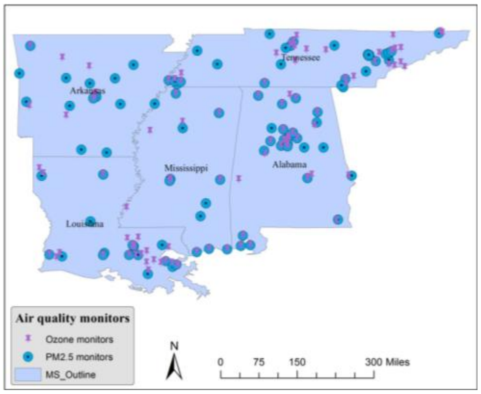


**Figure S1.** Air quality data for $O_3$ and $PM_{2.5}$ were obtained from ground monitoring stations located in the five states including Alabama, Arkansas, Louisiana, Mississippi, and Tennessee.

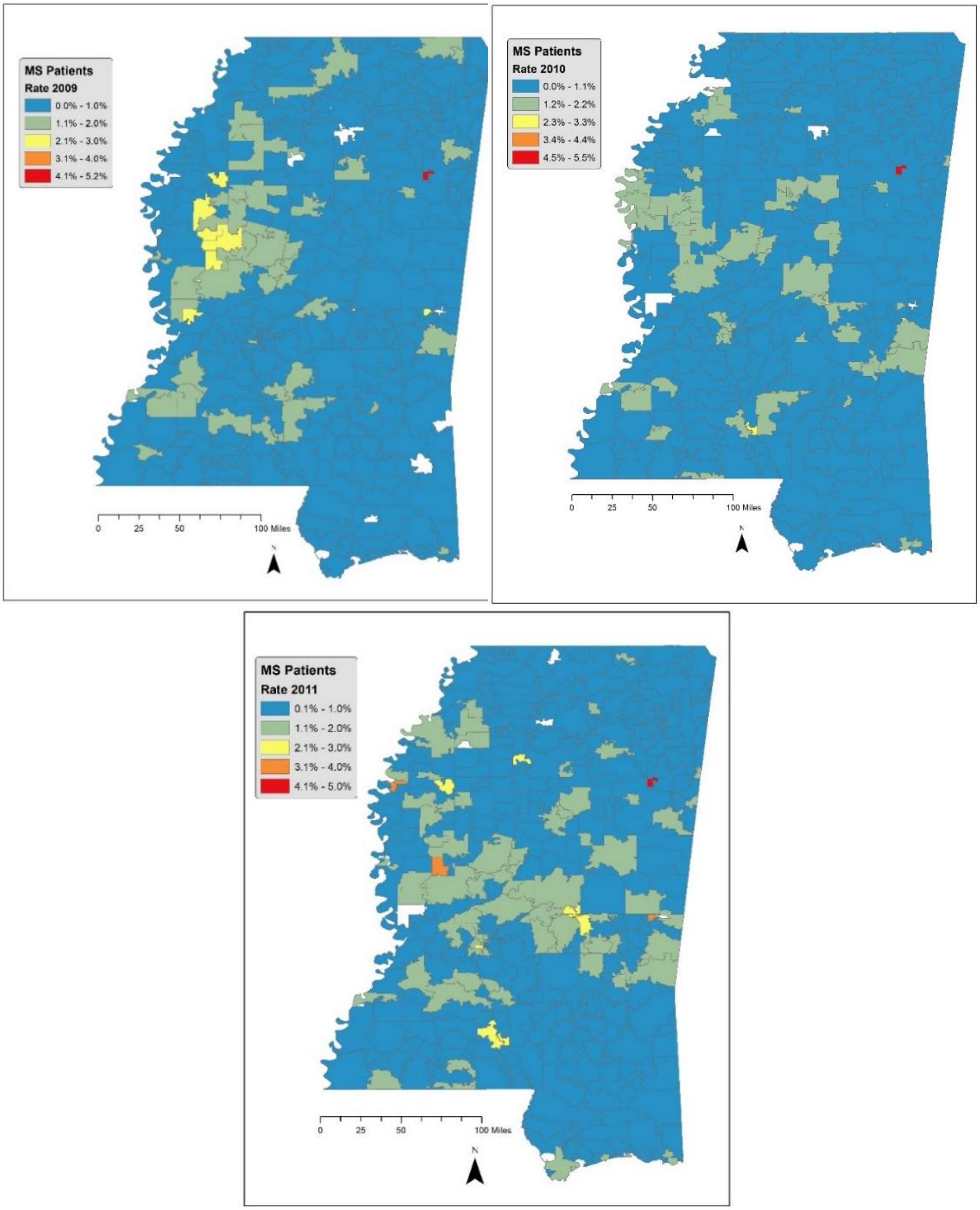


**Figure S2.** Spatiotemporal models of asthma rates by ZIP code 2009–2011.

**Table S1.** OLS Regression Analysis Results- Asthma Count as Dependent Variable.

| | Variable | Coefficient [a] | StdError | t-Statistic | Probability [b] | Robust_SE | Robust_t | Robust_Pr [b] | VIF [c] |
|---|---|---|---|---|---|---|---|---|---|
| **2009** | INTERCEPT | 117.813820 | 85.275 | 1.381575 | 0.167956 | 91.640475 | 1.285609 | 0.199399 | -------- |
| | AGE_75_84 | 0.037943 | 0.009071 | 4.182748 | 0.000041* | 0.015939 | 2.380511 | 0.017787* | 3.631406 |
| | FHH_CHILD | 0.002374 | 0.007835 | 0.303027 | 0.762050 | 0.015045 | 0.157814 | 0.874681 | 5.306596 |
| | COUNT_2010 | 0.112296 | 0.058531 | 1.918594 | 0.055811 | 0.183879 | 0.610706 | 0.541775 | 6.925643 |
| | COUNT_2008 | 0.389173 | 0.038087 | 10.217957 | 0.000000* | 0.134634 | 2.890594 | 0.004079* | 4.091101 |
| | PM_2.5_2009 | -11.615851 | 8.615158 | -1.348304 | 0.178404 | 9.250131 | -1.255750 | 0.210012 | 1.026822 |
| **OLS DIAGNOSTICS** | Input Features: 2009_Regression_Analysis_Data | | | | | Dependent Variable: COUNT_2009 | | | |
| | Number of Observations: 372 | | | | | Akaike's Information Criterion (AICc) [d]: 3690.421513 | | | |
| | Multiple R-Squared [d]: 0.727282 | | | | | Adjusted R-Squared [d]: 0.723556 | | | |
| | Joint F-Statistic [e]: 195.209146 | | | | | Prob(>F), (5,366) degrees of freedom: 0.000000* | | | |
| | Joint Wald Statistic [e]: 232.521698 | | | | | Prob(>chi-squared), (5) degrees of freedom: 0.000000* | | | |
| | Koenker (BP) Statistic [f]: 133.474588 | | | | | Prob(>chi-squared), (5) degrees of freedom: 0.000000* | | | |
| | Jarque-Bera Statistic [g]: 2578.441209 | | | | | Prob(>chi-squared), (2) degrees of freedom: 0.000000* | | | |
| **2010** | Variable | Coefficient [a] | StdError | t-Statistic | Probability [b] | Robust_SE | Robust_t | Robust_Pr [b] | VIF [c] |
| | INTERCEPT | 43.616479 | 34.371631 | 1.268967 | 0.205264 | 28.396249 | 1.535994 | 0.125417 | -------- |
| | AGE_75_84 | 0.016257 | 0.005217 | 3.116009 | 0.001989* | 0.007203 | 2.257121 | 0.024576* | 3.389893 |
| | FHH_CHILD | 0.001099 | 0.004840 | 0.227119 | 0.820458 | 0.009553 | 0.115080 | 0.908432 | 5.715478 |
| | COUNT_2011 | 0.754151 | 0.026488 | 28.471193 | 0.000000* | 0.046073 | 16.368765 | 0.000000* | 4.487035 |
| | COUNT_2009 | 0.160739 | 0.026172 | 6.141578 | 0.000000* | 0.063783 | 2.520080 | 0.012148* | 2.594331 |
| | PM_2.5_2010 | -4.014866 | 3.136900 | -1.279883 | 0.201403 | 2.582456 | -1.554670 | 0.120902 | 1.024073 |
| **OLS DIAGNOSTICS** | Input Features: 2010_Regression_Analysis_Data | | | | | Dependent Variable: COUNT_2010 | | | |
| | Number of Observations: 372 | | | | | Akaike's Information Criterion (AICc) [d]: 3304.476495 | | | |
| | Multiple R-Squared [d]: 0.935848 | | | | | Adjusted R-Squared [d]: 0.934972 | | | |
| | Joint F-Statistic [e]: 1067.838288 | | | | | Prob(>F), (5,366) degrees of freedom: 0.000000* | | | |
| | Joint Wald Statistic [e]: 2880.892494 | | | | | Prob(>chi-squared), (5) degrees of freedom: 0.000000* | | | |
| | Koenker (BP) Statistic [f]: 38.561430 | | | | | Prob(>chi-squared), (5) degrees of freedom: 0.000000* | | | |
| | Jarque-Bera Statistic [g]: 22970.196414 | | | | | Prob(>chi-squared), (2) degrees of freedom: 0.000000* | | | |
| **2011** | Variable | Coefficient [a] | StdError | t-Statistic | Probability [b] | Robust_SE | Robust_t | Robust_Pr [b] | VIF [c] |
| | INTERCEPT | -83.081761 | 32.712538 | -2.539753 | 0.011496* | 24.314921 | -3.416904 | 0.000718* | -------- |
| | AGE_75_84 | -0.012354 | 0.005767 | -2.142353 | 0.032811* | 0.009907 | -1.247038 | 0.213183 | 3.460515 |
| | FHH_CHILD | 0.029315 | 0.005043 | 5.812576 | 0.000000* | 0.013555 | 2.162723 | 0.031195* | 5.184487 |
| | COUNT_2010 | 0.894310 | 0.027997 | 31.943004 | 0.000000* | 0.131777 | 6.786553 | 0.000000* | 3.736648 |
| | PM_2.5_2011 | 7.996147 | 3.081967 | 2.594495 | 0.009846* | 2.303258 | 3.471669 | 0.000592* | 1.045064 |
| **OLS DIAGNOSTICS** | Input Features: 2011_Regression_Analysis_Data | | | | | Dependent Variable: COUNT_2011 | | | |
| | Number of Observations: 372 | | | | | Akaike's Information Criterion (AICc) [d]: 3370.236851 | | | |
| | Multiple R-Squared [d]: 0.931321 | | | | | Adjusted R-Squared [d]: 0.930572 | | | |
| | Joint F-Statistic [e]: 1244.171060 | | | | | Prob(>F), (4,367) degrees of freedom: 0.000000* | | | |
| | Joint Wald Statistic [e]: 1474.596958 | | | | | Prob(>chi-squared), (4) degrees of freedom: 0.000000* | | | |
| | Koenker (BP) Statistic [f]: 154.180031 | | | | | Prob(>chi-squared), (4) degrees of freedom: 0.000000* | | | |
| | Jarque-Bera Statistic [g]: 9075.539496 | | | | | Prob(>chi-squared), (2) degrees of freedom: 0.000000* | | | |

**Table S2.** OLS Regression Analysis Results -$PM_{2.5}$ as Dependent Variable.

| | Variable | Coefficient [a] | StdError | t-Statistic | Probability [b] | Robust_SE | Robust_t | Robust_Pr [b] | VIF [c] |
|---|---|---|---|---|---|---|---|---|---|
| **2011** | INTERCEPT | 10.226941 | 0.086157 | 118.7016 | 0.000000* | 0.094680 | 108.016365 | 0.000000* | -------- |
| | ASTHMA PER CAPITA | 0.242101 | 0.040142 | 6.031114 | 0.000000* | 0.035371 | 6.844706 | 0.000000* | 1.074500 |
| | POVERTY RATE | 0.466070 | 0.170201 | 2.738355 | 0.006474* | 0.162134 | 2.874598 | 0.004285* | 1.0665 |
| | ADULTS 75UP PER_CAPITA | 0.012399 | 0.012570 | 0.986408 | 0.324570 | 0.013042 | 0.950684 | 0.342376 | 1.010506 |

| | | |
|---|---|---|
| OLS DIAGNOSTICS | Input Features: 2011_Regression_Analysis_Data | Dependent Variable: PM_EXT2011 |
| | Number of Observations: 372 | Akaike's Information Criterion (AICc) [d]: 296.160204 |
| | Multiple R-Squared [d]: 0.135559 | Adjusted R-Squared [d]: 0.128512 |
| | Joint F-Statistic [e]: 19.236276 | Prob(>F), (3,368) degrees of freedom: 0.000000* |
| | Joint Wald Statistic [e]: 71.678747 | Prob(>chi-squared), (3) degrees of freedom: 0.000000* |
| | Koenker (BP) Statistic [f]: 26.373684 | Prob(>chi-squared), (3) degrees of freedom: 0.000008* |
| | Jarque-Bera Statistic [g]: 7.749826 | Prob(>chi-squared), (2) degrees of freedom: 0.020756* |

AIMS Press

© 2018 the Author(s), licensee AIMS Press. This is an open access article distributed under the terms of the Creative Commons Attribution License (http://creativecommons.org/licenses/by/4.0)
